# Harnessing Paleohydrologic Modeling to Solve a Prehistoric Mystery

**DOI:** 10.1038/s41598-019-52761-x

**Published:** 2019-11-08

**Authors:** Yehuda Levy, Nigel A. Goring-Morris, Yoseph Yechieli, Avihu Burg, Haim Gvirtzman

**Affiliations:** 1 Institute of Earth Sciences, The Hebrew University of Jerusalem, Edmond J. Safra Campus, Givat Ram, Jerusalem 91904 Israel; 2 Institute of Archaeology, The Hebrew University of Jerusalem, Jerusalem, 91905 Israel; 3 Geological Survey of Israel, 32 Yeshayahu Leibowitz St., Jerusalem, 9692100 Israel; 4 Zuckerberg Institute for Water Research, Ben-Gurion University, Sede Boqer Campus, Sede-Boker, 8499000 Israel

**Keywords:** Archaeology, Hydrology

## Abstract

A riddle arises at the Epipaleolithic and Neolithic sites that dot the lower Jordan Valley. The area has no water resources yet it has long been a focus of inquiry into the transition from mobile hunter-gatherer to sedentary agriculture-based cultures. How then is there such clear evidence of life here, and particularly at such a critical moment in human evolution? Keen to unravel this conundrum, a numerical hydrological model was devised to simulate the groundwater flow field within the Eastern Aquifer of the Judea and Samaria Mountains during the transition from the last glacial to the current interglacial. The model exhibits a range of groundwater flow regimes that prevailed in the past, demonstrating that there was once much larger groundwater discharge at these sites.

## Introduction

This study marks a rare and rewarding collaboration between hydrologists and archeologists. Together we were puzzled by the contradiction between the prehistoric archeology of the lower Jordan Valley (Supplementary Table 1 and its references), with its evidence of agriculture, and the apparent absence of freshwater resources. The early settlements must have had access to water^1–3^, especially those that shifted from being mobile hunter-gatherers to sedentary agriculture-based cultures^4,5^, but with neither springs nor fresh surface water in sight it is unclear how they irrigated their fields, or indeed survived.

Computerized models of groundwater flow and solute transport are effective modern tools for planners and engineers engaged in the exploitation of water resources and remediation of contaminated aquifers^6,7^. These models are also used to explain geological phenomena, such as the formation of economic ores, migration of hydrocarbons within rock formations into traps and transportation of geothermal energy^8^. Here, we employ groundwater flow numerical modeling to tackle our prehistoric riddle.

## Jordan Valley Prehistoric Settlements

**Figure 1:**
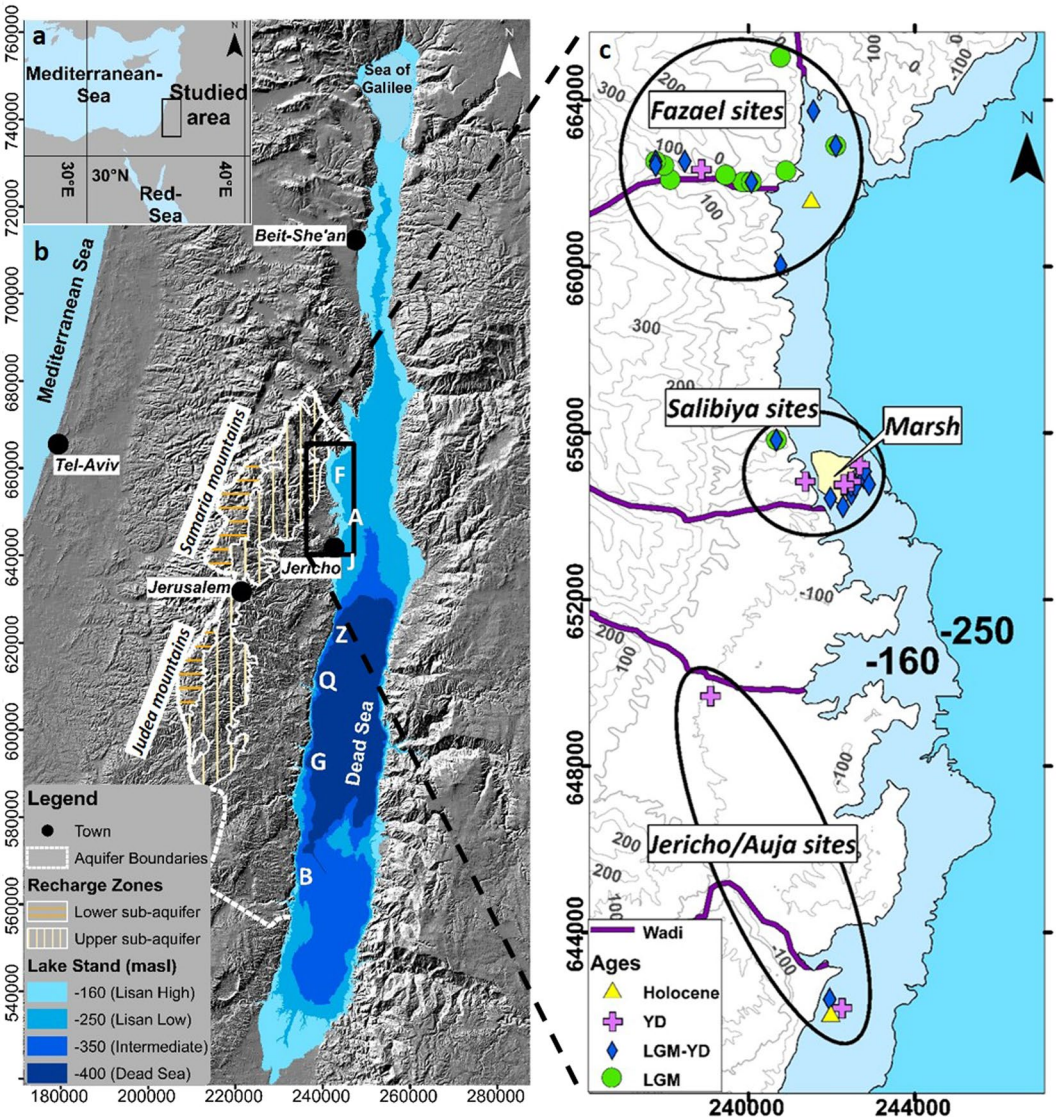
Studied area. (**a**) A location map. (**b**) The boundaries of the Eastern Mountain Aquifer are drawn over a shaded relief map^46^, including the recharge areas into the two sub-aquifers, as well as the seven natural discharge zones in the Jordan-Dead Sea rift valley: F - Fazael, A - Auja, J - Jericho, Z – Zuqim, Q - Qane-Samar, G - Ein-Gedi and B -Boqeq. The lake that existed within the rift valley is drawn under four lake stands: high Lake Lisan at −160 mbsl, low Lake Lisan at −250 mbsl, Transition Lake at −350 mbsl, and the Dead Sea at −400 mbsl. (**c**) Zooming into the lower Jordan rift valley, where three groups of prehistoric settlements were identified. All were located adjacent to Lake Lisan or Transition Lake and had a continued presence over four climatological periods (Supplementary Table 1). Although today no springs exist near these settlements, during the pre-historic higher lake stands, considerable quantities of groundwater discharged there, sometimes creating a freshwater marsh. All coordinates are of the Israeli Transverse Mercator (ITM) system.

Prehistoric archeologists identified three centers of early cultivation – central Mexico, the middle Yangtze River in China, and the Mediterranean Levant. All were loci of a pivotal step in human evolution, the shift from hunter-gatherer to agriculture-based cultures, known as the Agricultural Revolution^9,10^, though the best-recorded sequence of this shift is found in the last of the three: in the Levantine corridor that stretches from the southern flanks of the Taurus Mountains in Turkey to the Sinai Peninsula in Egypt. More specifically, one of the best sequences exists in the lower Jordan Valley (Fig. 1)^9,11,12^. The emergence of sedentary communities in the Levant occurred between 15,000 and 10,000 cal years BP. The process involved two major archaeological entities, the Natufians and the earliest Neolithic (termed Pre-Pottery Neolithic A - PPNA). These were followed by the cultural entities of the Pre-Pottery Neolithic B period (PPNB) with extensive evidence of sedentary village communities as well as domesticates (both plants and animals). The Natufians were secondary foragers and, possibly, the earliest farmers on Earth^9,13^.

All 45 prehistoric sites of the lower Jordan Valley are presented in Fig. 1c, along with their corresponding ages (Supplementary Table 1). Existing before, during and after the Agricultural Revolution, they were concentrated in three locations: Fazael in the north, Salibiya in the center, and Jericho/Auja in the south. They correlate with a sequence of four climate stages, as follows: Thirteen sites from the Last Glacial Maximum^14,15^ (LGM), dated up to ca. 18.5 cal ka BP, were documented in the northern Fazael area; 19 sites from the Kabarian and Natufian cultures, dated from the end of LGM up until the Younger Dryas (YD) event, a period that saw the Earth’s climate warmed gradually^16,17^ (ca. 18.5-12.5 cal ka BP), were documented in the Fazael and Salibiya areas; additional nine sites are Pre-Pottery Neolithic, which existed during the YD (ca. 12-11 cal ka BP), over the three locations; and finally, four sites that existed in the Holocene (from ca. 11 cal ka BP), when the Earth’s climate warmed, were documented in the southern Jericho/Auja area. The spatial and temporal distribution of these prehistoric sites demonstrate southward migration of the local populations with time.

## Jordan Valley lakes

Lakes in the Jordan Valley expanded and shrank during the Quaternary due to climate changes, expressed in variations in rainfall amounts^18,19^ (Supplementary Fig. 1). During the last glacial (70 ka BP −18 cal ka BP), Lake Lisan extended between 160 to 250 km along the valley, and its level fluctuated between −250 to −160 meters below mean sea level (mbsl)^20,21^, respectively. During the Holocene, two small lakes existed in this area: the freshwater Sea of Galilee in the north, whose level stands at −210 mbsl, and the hyper-saline Dead Sea in the south, at about −400 mbsl (Fig. 1b). Following the end of the last glacial period (ca. 18 cal ka BP), the Earth’s climate warmed gradually^14,15,20^, but that trend was interrupted for about 1,000 years during the YD (ca. 12-11 cal ka BP), when the climate in the Levant reverted to a short cold state before temperatures began to rise again^16,22^. In the intervening interval (ca. 18-9 cal ka BP), an intermediate lake existed in the Jordan Valley – we term it Transition Lake – whose level was initially ca. −250 and later −420 mbsl^17,23,24^. During this interval, including the YD, the prehistoric settlements were located on the shores of Transition Lake, but it cannot have served as their water source because, like Lake Lisan, it was not a freshwater lake; the salt concentration of both lakes was between 3–5 times that of the oceans (or even more for the ~−400 mbsl lake), or about one third to half of today’s Dead Sea salinity which is 342 gr/l (total dissolved solid)^25,26^. So, what was the water source?

## Regional Hydrogeology

Rain falling on the central range of the Judea and Samaria Mountains (Fig. 1) permeates the regional aquifer (after losing for evapotranspiration and runoff about two thirds), flowing westward and eastward, and discharges through springs on the Mediterranean coastal plain and along the Jordan Rift Valley. The eastward flow occurs in the Eastern Mountain Aquifer of Judea and Samaria (EMA). Past studies^27–32^ have shown that the EMA is karstic, and groundwater is stored in the dolomite and limestone rocks of the Judea Group (Albian-Turonian age). The groundwater flows from the EMA into the rift filling units in the Jordan Valley and discharges mainly through the Zuqim, Qane and Samar springs near the Dead Sea (Fig. 1b). The total EMA discharge (including direct discharge to the Dead Sea) is ca. 150 million cubic meters per year (mcm/y), (supplementary Fig. 4).

The EMA is divided vertically into two sub-aquifers with an aquitard layer in between. The average gradient of the water table toward the Jordan Valley and the Dead Sea is steep (~4%), forcing considerable fresh groundwater flow toward the outlets; the flow is complex and is affected by the intricate geological structure (Supplementary Fig. 2). The Dead Sea, located at the lowest elevation in the rift valley, serves as the deepest drainage basin of the EMA. Presently, the lake level is −433 mbsl (in 2019, Israel Water Authority). Due to anthropogenic water diversion in the last few decades,the Dead Sea level is falling at a rate of ~1 m/year^33^. It is predicted that it will continue to drop in the coming ~200 years, additional 100–150 m, before reaching a new steady state^34,35^.

## Paleo-hydrological Modeling

**Figure 2:**
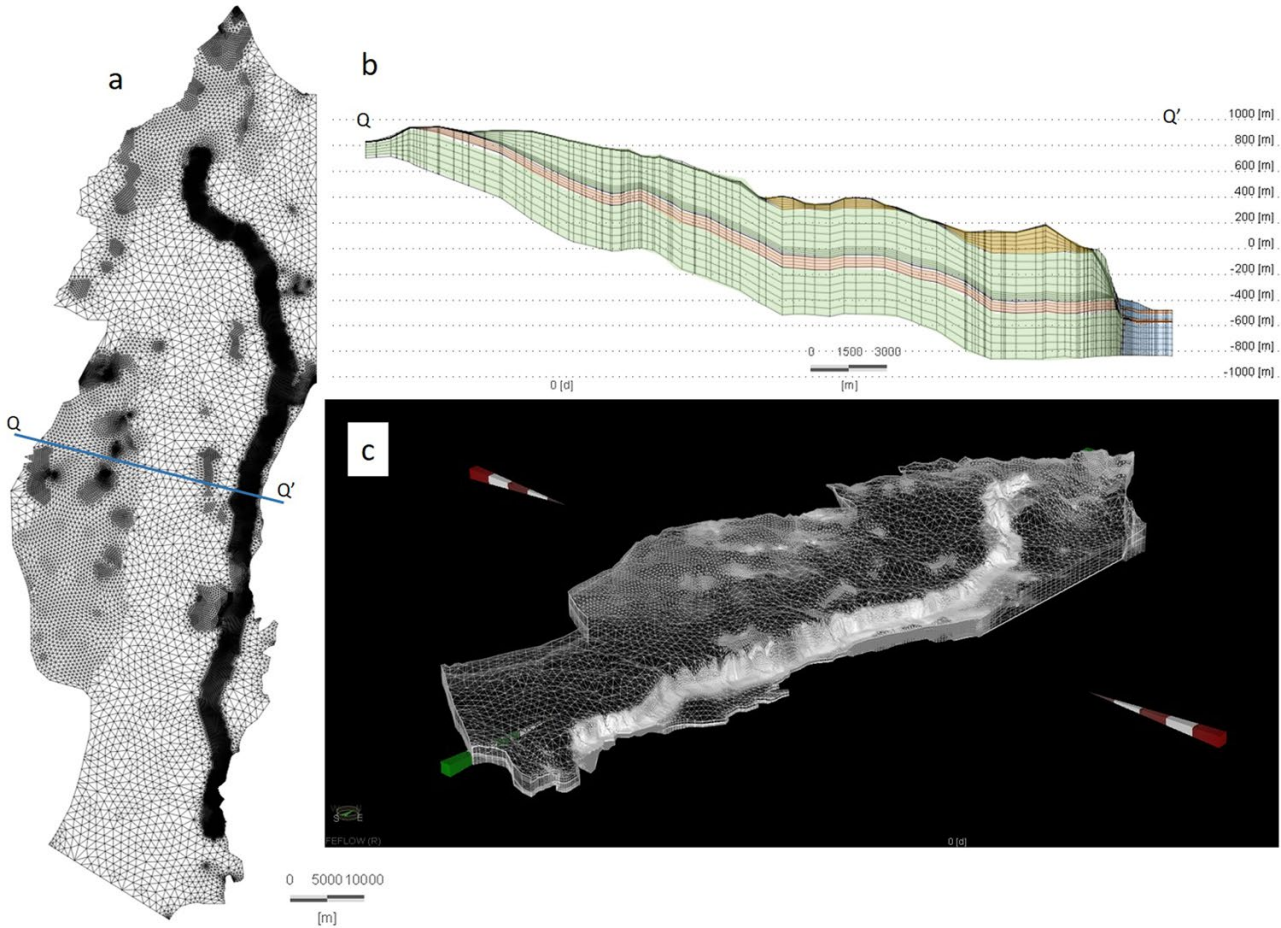
Mesh of the numerical model, prepared with FEFLOW software. (**a**) Map view with the mesh and boundaries (as in Fig. 1 and Supplementary Fig. 1). (**b**) Q-Q’ cross-section (location are marked on Fig. 2a). (**c**) Three-dimensional view of the mesh.

**Figure 3:**
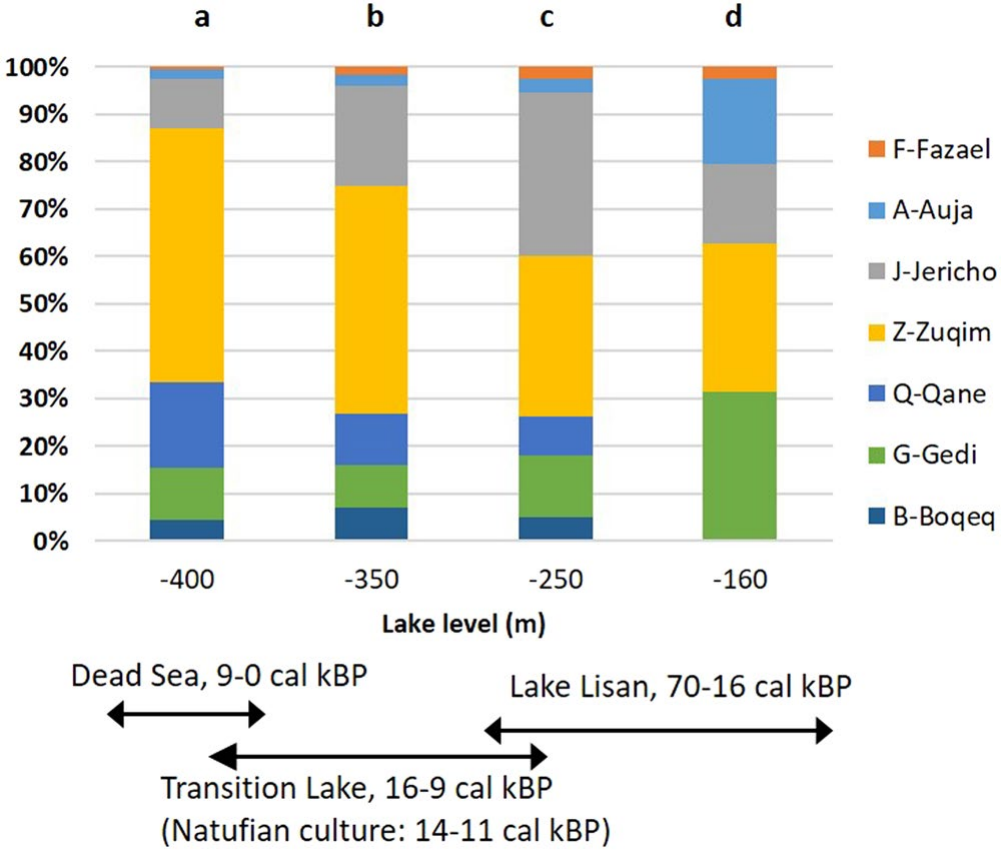
The relative amount of groundwater discharges at the seven discharge zones (locations in Fig. 1b). Each discharge amount is calculated by the numerical model for each of the four stands of the lake. The spreading areas of these lakes are shown in Fig. 1b. Currently (**a**), at ~−400 mbsl, a major amount of groundwater emerges at the Zuqim spring zone, but under higher lake level stands (**b**–**d**), the Zuqim discharge decreased and the discharges at the northern spring zones increased. The total discharge amount equals ~150 million cubic meters per year (mcm/y) at present, but during the glacial period it might have risen to up to ~200 mcm/y.

**Figure 4:**
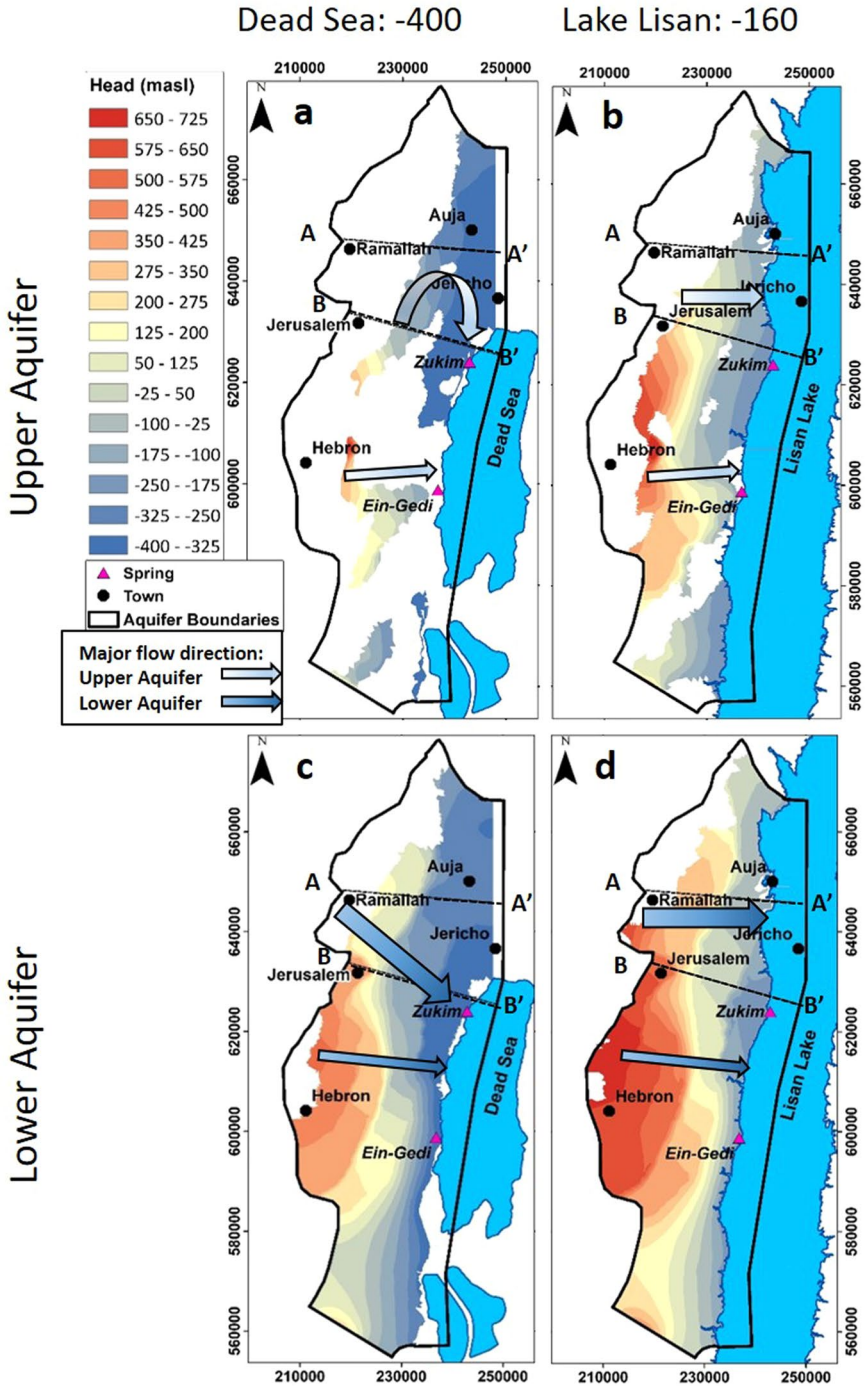
Maps of water table elevations for the upper (**a**,**b**) and lower (**c**,**d**) sub-aquifers. Each is calculated numerically for two given lake stands: −400 mbsl for the current Dead Sea and −160 mbsl for Lake Lisan highest stand. Under the Dead Sea conditions, the upper sub-aquifer is partly dry. The lines represent locations of the hydrogeological cross sections shown in Fig. 5. Major flow trajectories (arrows) demonstrate the shift of the groundwater flow direction from the Samaria Mountains.

Since the lakes in the Jordan Valley serves as the EMA’s drainage basin, we hypothesized that a considerable change in lake level will affect the groundwater flow field. Thus, we applied our hydrological numerical model to examine this hypothesis (Fig. 2, Supplementary Figs 2–6, Tables 2–4). In the first stage, the model was calibrated using measured spring discharge rates and observed water table elevations at >100 wells throughout the aquifer area. The model reconstructed the current groundwater flow field (Figs 3a and 4a,c) and provided information about hydraulic properties of the various rock layers. In the second stage, we used this calibrated model to simulate the groundwater flow field when Lake Lisan and Transition Lake covered the Jordan Valley (Figs 3b–d, 4b,d and Supplementary Fig. 7). This time it presented a flow regime in the aquifer toward the lake, with levels of: −350, −250, −160 mbsl (Figs 3–4 and Supplementary Fig. 7). These surprising results show that as the lake level decreases, it is followed by a coeval southward shift in the flow system; namely, smaller quantities of groundwater flow toward the Jordan Valley, north of the Dead Sea. The model results indicate (Fig. 3) that currently >50% of the rainwater amount penetrating the aquifer, discharges through the Zuqim Spring (Fig. 1), >30% discharges through other springs along the Dead Sea shore, and only ~10% flow toward the northern discharge zones (and no water discharges at the prehistoric sites). However, a major change is observed for Lake Lisan and Transition Lake intervals; just 30% of the infiltrating rain discharged at Zuqim Spring and >30% discharged toward the northern zones (Figs 1 and 3). This indicates that the lower Jordan Valley, well known for its aridity, once boasted freshwater springs, streams and swamps.

These insights are buttressed by independent evidence: tufa, or travertine (a carbonate spring deposit, Supplementary Fig. 8), is found adjacent to the prehistoric sites, along the reconstructed shores of Lake Lisan and Transition Lake (Fig. 1c). Some of these deposits were dated in archeological studies, to the intervals of higher lake levels^3,36,37^, attesting to the presence of paleo-springs. Quantifying the discharge of these springs is now possible with the numerical modeling (Fig. 3).

## Changing The Groundwater Flow Field

The change in the groundwater flow field in the EMA derives from two core factors: (1) changes in the “boundary conditions”, namely the location of the lowest draining level, and (2) blockages in the flow toward the Dead Sea in the deep sub-aquifer. Currently, given the Dead Sea as the lowest level, groundwater in the Judean Mountains flows eastward. However, from the Samarian Mountains, which is the main recharge contributor (Supplementary Fig. 4), groundwater flows southeastward along the steepest hydraulic gradient toward the Dead Sea (Figs 1 and 4). On the other hand, now that we can better assess the conditions at Lake Lisan and Transition Lake, we see that groundwater from the Samarian Mountains once flowed eastward, i.e., along the shortest path to the paleo-springs in the lower Jordan Valley (Figs 1, 4, Supplementary Figs 4 and 9).

**Figure 5:**
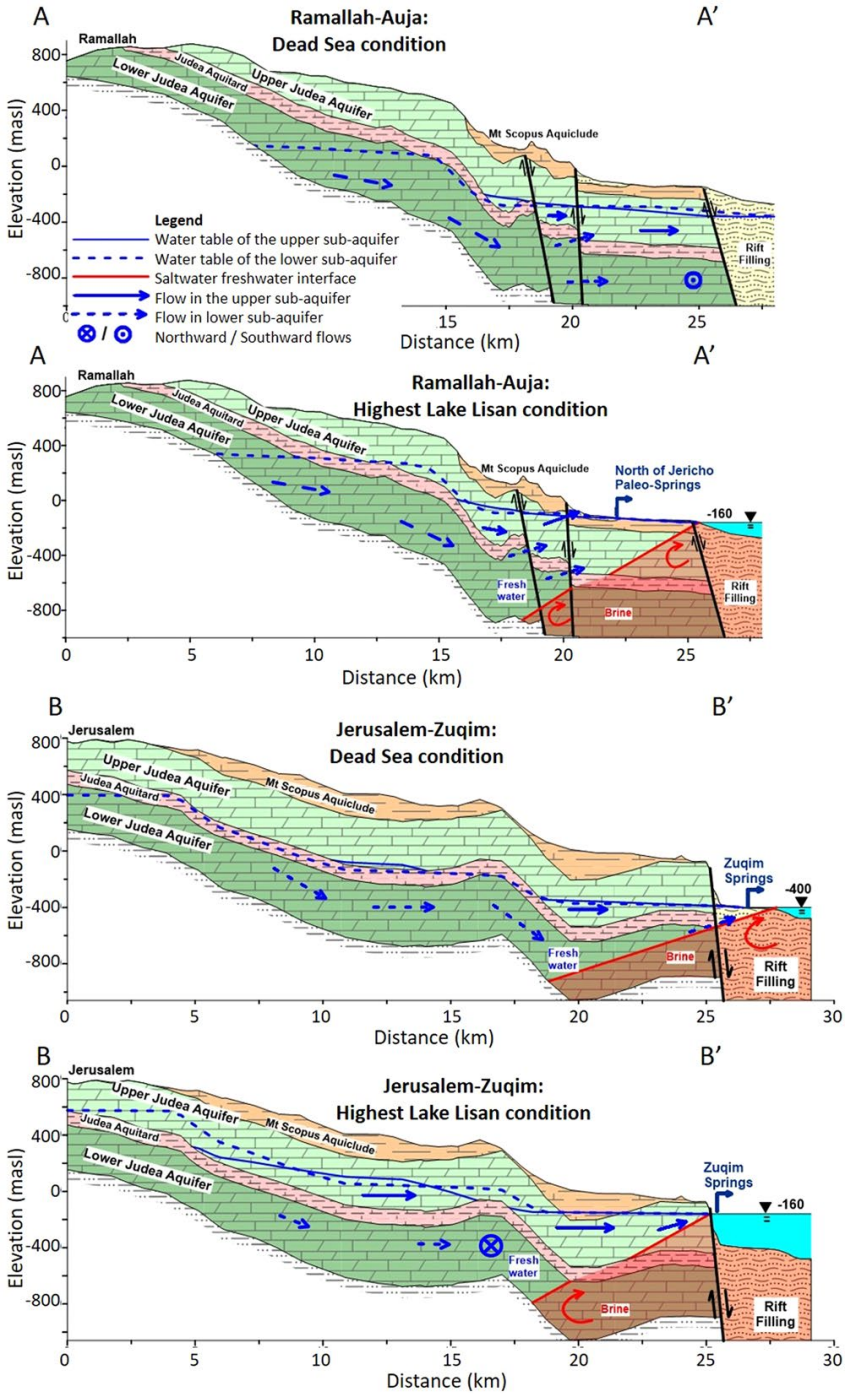
Two east-west hydrogeological cross sections (locations are shown in Fig. 4). Each is calculated numerically for the two lake stands. Water table elevations, flow lines in the upper and lower sub-aquifers and the interfaces between fresh groundwater and saline lake water are marked. Due to the geological structure, the high lake level is associated with blocking flows from the lower sub-aquifer toward the Zuqim spring zone, thereby pushing the discharge northward to Jericho-Fazael-Auja zones.

The blocking flow in the deep sub-aquifer of groundwater flow towards the Dead Sea springs is the outcome of the high lake level that formed a higher interface between the freshwater of the aquifer and the saltwater of the lake (Fig. 5 and Supplementary Fig. 9). Such an interface exists in all coastal aquifers, causing the lower-density fresh groundwater to “float” over the high-density saline seawater. Due to the specific and complicated geological structure of the EMA, and given the high lake level, blockage of the groundwater flow towards the Zuqim springs in the lower sub-aquifer enhanced the discharge at more northern discharge zones (Fig. 5). Thus, in addition to the groundwater originating from the Samarian Mountains, some excess groundwater in the Judea Mountains diverted northeastward to the paleo-springs of the lower Jordan Valley.

## Discussion

Numerical modeling of the groundwater flow in the EMA proved to be challenging. Here we outline some of the challenges, as well as limitations inherent in the modeling and the achievements it yielded in this study.

Not only does the basin span a vast area and reach great depth, it is also characterized by a complicated geological structure. It comprises more than 10 rock formations with vastly different hydraulic properties and the rock layers are folded and faulted, resulting in severe horizontal and vertical heterogeneity. These geological structures required the use of three-dimensional (3D) description of such a heterogeneous basin requires a detailed, fine mesh. In 2D flow simulations, spring migration would not have been observed. The 3D model was successful, and its results reliable (Supplementary Figs 2–9 and Tables 2 and 3). The model’s limitations were tackled by introducing several assumptions, especially with regard to the grouping of several rock formations into fewer hydro-stratigraphic units.

Other constraints pertaining to the simulation of the solute transport and the interface between the fresh groundwater and the hypersaline lake water. The mesh for the flow modeling includes several million elements, whose average area is tens of thousands of square meters and average depth is tens of meters, but the transport simulations would require at least three orders of magnitude of smaller elements, which is unachievable. Therefore, the location of the interface is determined according to the Ghyben^38^–Herzberg^39^ approximation, using the salinities of the two water bodies. This approximation is satisfactory, as indeed was found in several boreholes near the Dead Sea where the depth of the interface was according to the Ghyben–Herzberg approximation^40,41^.

Climate changes between glacial and inter-glacial periods can dramatically affect rainfall amounts and evaporation rates (and thereby the recharge amounts). Therefore, the sensitivity of the modeling results (the flow regime) related to recharge amounts was analyzed. The net rainwater recharge (and discharge under steady-state conditions) during glacial is estimated as 33% higher than present value. Unexpectedly, it was found that the relative discharge amounts among the discharge zones were constant, under all lake levels condition, regardless the recharge amount. Thus, the vertical axis in Fig. 3 refers to relative (not absolute) values. Based on the sensitivity tests, it is clear that the locations of the discharge zones depend only on the elevation of the base level and not on the recharge values.

## Conclusions

The numerical modeling with its challenges and limitations indicates with great confidence that the large springs discharging today along the Dead Sea experienced lower discharge when Lake Lisan level in the Jordan Valley was higher. This indicates that during the last glacial, when Lake Lisan extended across the Jordan Valley (70–18 cal kBP), large springs with significantly higher groundwater discharges existed north of the Dead Sea, in the lower Jordan Valley, where they do not exist today. A total amount of 30–40 mcm/y of freshwater enabled to transform this dry environment to become habitable for the hunter-gatherer groups. Later, when Transition Lake with its fluctuating level spread in the Jordan Valley, the springs north of the Dead Sea supplied 10–15 mcm/y. These springs generated freshwater swamps adjacent to the saline lake, creating a livable environment for sedentary communities and early cultivation of the Natufian and PPNA cultures. The general southward migration of the Epipaleolithic and Neolithic populations (Fig. 1c, Supplementary Table 1) followed the shrinking of the Jordan Valley lakes and the migration of the paleo-springs through which the EMA was discharged.

## Methods

In this study, we used a computerized hydrological model to describe the flow field in the Eastern Mountain Aquifer of the Judea and Samaria Mountains. Our model is based on the groundwater flow equations. In other words, it ensures that the water input and output – i.e., the net amounts of rainfall that permeate the aquifer (input) and of groundwater emerging in the springs and flowing in the subsurface into the Jordan and the Dead Sea (output) – are equal. The model also calculates the rate of groundwater flow in the various hydrogeological units according to their hydraulic properties, namely, their conductivities. It is calibrated by equalizing the calculated and measured water levels in wells, and by equalizing the measured and calculated groundwater quantities discharging in springs. This enables us to reconstruct past groundwater flow fields under the conditions of higher lake levels in the Jordan Valley.

To devise our model, geological, lithological and hydrological data from all available sources were collected. Based on a structural map of the top upper sub-aquifer, the Top Judea Group^42^, structural maps of several other horizons were drafted using lithological data from 106 boreholes and outcrops scattered around the EMA. The new maps outline the tops and bottoms of major hydro-stratigraphic units: the top of the Moza Formation (the base of the Upper Aquifer and the top of the Aquitard), the base of the Beit Meir Formation (the base of Aquitard and the top of the Lower Aquifer) and the base of the Kfira Formation (the base of the Lower Aquifer) (see the stratigraphic table of the geological units in Supplementary Fig. 3). The 3D model domain was then set up using FEFLOW software^43^. This software enables numerical modeling of groundwater, using the finite element method, for solving the flow equation of groundwater flow. The EMA model includes 161501 elements per layer, 30 layers (slices), maximal element area: 1.25 Km^2^, minimal element area: 1 m^2^, mean element area: 19640 m^2^ and standard deviation of the element area is 77755 m^2^ (Fig. 2).

The numerical flow model receives the recharge values as input. A precipitation map was prepared by spatial interpolation (Kriging method) of average precipitation data (Supplementary Fig. 4) from 37 rain gauges of the Israel Meteorological Service. Recharge equations (distribution of precipitation minus evaporation) of the EMA were developed in a previous study^30^, depending on the annual precipitation quantity (Eqs 1–3). Where the average annual precipitation (P) is more than 650 mm, the average recharge (R) is:

1 $$R=0.8\cdot (P-360)$$

Where the average annual precipitation is less than 650 mm and more than 300 mm, the average annual recharge is:

2 $$R=0.534\cdot (P-216)$$

Finally, where the average annual precipitation is less than 300 mm, the average annual recharge is:

3 $$R=0.15\cdot P$$

We used these equations to calculate a recharge map from the precipitation map (Supplementary Fig. 4). The recharge occurs only on the dolomite and limestone outcrops of the Judea Group, and is prevented where chalk and marl rocks of the overlying Mt. Scopus Group are exposed (see Supplementary Fig. 2).

The boundary conditions of the flow modeling are hydraulic head at the lake shoreline (−400 mbsl for the Dead Sea and higher for Lake Lisan and Transition Lake), fluid transfer at the springs near the shoreline, and recharge flux on top of the model. No flow boundary condition applied at the bottom of the model, its western and northern sides, and at the eastern side of the model below lake level. At the southern side of the model, a flow boundary condition is defined to represent the actual water flux from the Negev (about 5 MCM/y).

Calibration was conducted under steady state conditions, without the effect of pumping in wells. The water table data are from ~1970 to ~1980, prior to the pumping in the region. Therefore, the Dead Sea level in the calibration is −400 mbsl and not the 2019 level of −433 mbsl. The hydraulic conductivities of the two sub-aquifers and the aquitard (Supplementary Fig. 5) and the transfer rate coefficient of the spring outlets (0.8–0.008 1/day) were calibrated. This calibration was performed manually by trial-and-error runs, in which the error between observed and calculated hydraulic head of the water wells was minimized. The resulted mean absolute error is less than 2.5% of the difference between the highest and lowest hydraulic head of the EMA (Supplementary Fig. 6, Tables 2 and 3). The anisotropy of the hydraulic conductivity (vertical/horizontal) was set to be 0.1. Zoning of equal hydraulic conductivity areas throughout the aquifer is based on geological properties of the EMA: where the geological layers are folded, the hydraulic conductivities are lower^44^. In some significant faults, higher hydraulic conductivity was set in the aquitard, so water can flow between the upper and lower sub-aquifers. The calibrated hydraulic conductivities are similar (the same orders of magnitude) to those of the nearby Yarqon-Taninim aquifer, which is composed of the same rocks^45^, at the westward basin of the Judea and Samaria Mountains.

The calibrated numerical flow model was employed later to simulate the flow regime in the EMA under the conditions of higher lake levels. Numerical simulations were run under the following lake levels: −350, −250, −200, −160 mbsl (Supplementary Figs 7 and 9). Appropriate hydraulic head boundary conditions were set to the model for each of the lake level simulations. The discharge spring zones (Figs 1 and 3) defined by geographic discretion and discharge quantities through these zones were then examined.

The freshwater saline-water interface locations are calculated according to the Ghyben-Herzberg ratio^38,39^ at each of the cross sections of Fig. 5 and Supplementary Fig. 9. The density of the current Dead Sea water (−400 mbsl) is set to be 1.24 kg/liter^33^, and the density of Lake Lisan and Transition Lake waters (−350, −250 and −160 mbsl) is set to be 1.12 kg/liter^25,26^.

It should be noted that some small springs existing today in the lower Jordan Valley (e.g., Fazael springs) were excluded in the EMA modeling. That is because they discharge up to hundreds of thousands cubic meters per year from a perched aquifer only during wet winters. The numerical model takes into account only stable and permanent regional springs that discharge larger amounts, of at least two orders of magnitude, from the regional aquifer.

## Supplementary information

Supplementary Informations
