# Supplementary material for: Harnessing Paleohydrologic Modeling to Solve a Prehistoric Mystery
Source: Sci Rep. 2019 Nov 8;9:16349. doi: 10.1038/s41598-019-52761-x (PMC6841701; doi:10.1038/s41598-019-52761-x)
Supplement: Supplementary file 1 — Supplementary Informations [file 41598_2019_52761_MOESM1_ESM.pdf]

Supplementary Information for:

Harnessing Paleohydrologic Modeling to Solve a Prehistoric Mystery

Yehuda Levy[1], Nigel A. Goring-Morris[2], Yoseph Yechieli[3,4], Avihu Burg[3] and Haim Gvirtzman[1]

[1]Institute of Earth Sciences, The Hebrew University of Jerusalem, Edmond J. Safra Campus, Givat Ram, Jerusalem 91904, Israel.

[2]Institute of Archaeology, The Hebrew University of Jerusalem, 91905, Israel.

[3]Geological Survey of Israel, 32 Yeshayahu Leibowitz St., Jerusalem 9692100, Israel.

[4]Zuckerberg Institute for Water Research, Ben-Gurion University, Sede Boqer Campus, 8499000, Israel.

Correspondence and requests for materials should be addressed to Y.L. (email: yehuda.levy1@mail.huji.ac.il)

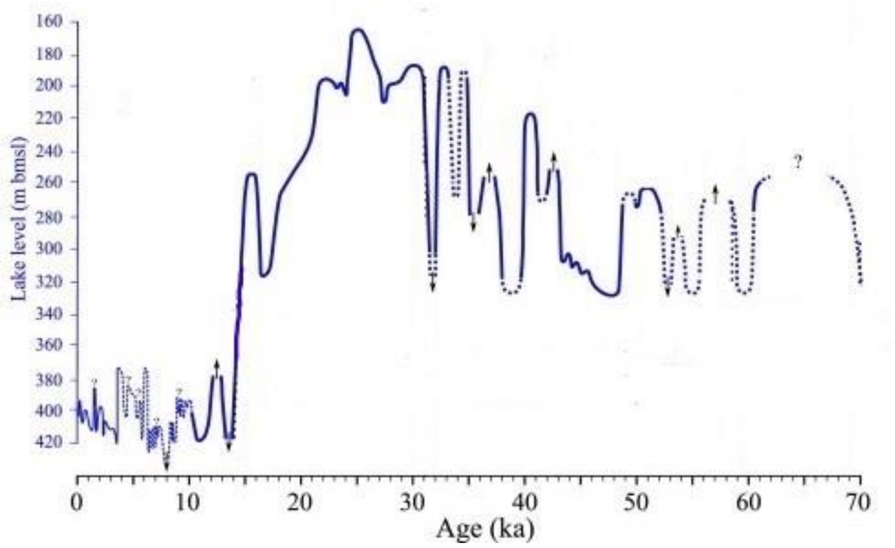

Supplementary Fig. 1. Jordan Valley Lake levels. Levels of Lake Lisan (70-16 cal kBP), Transition Lake (16-9 cal kBP) and the Dead Sea (9-0 cal kBP)[21].

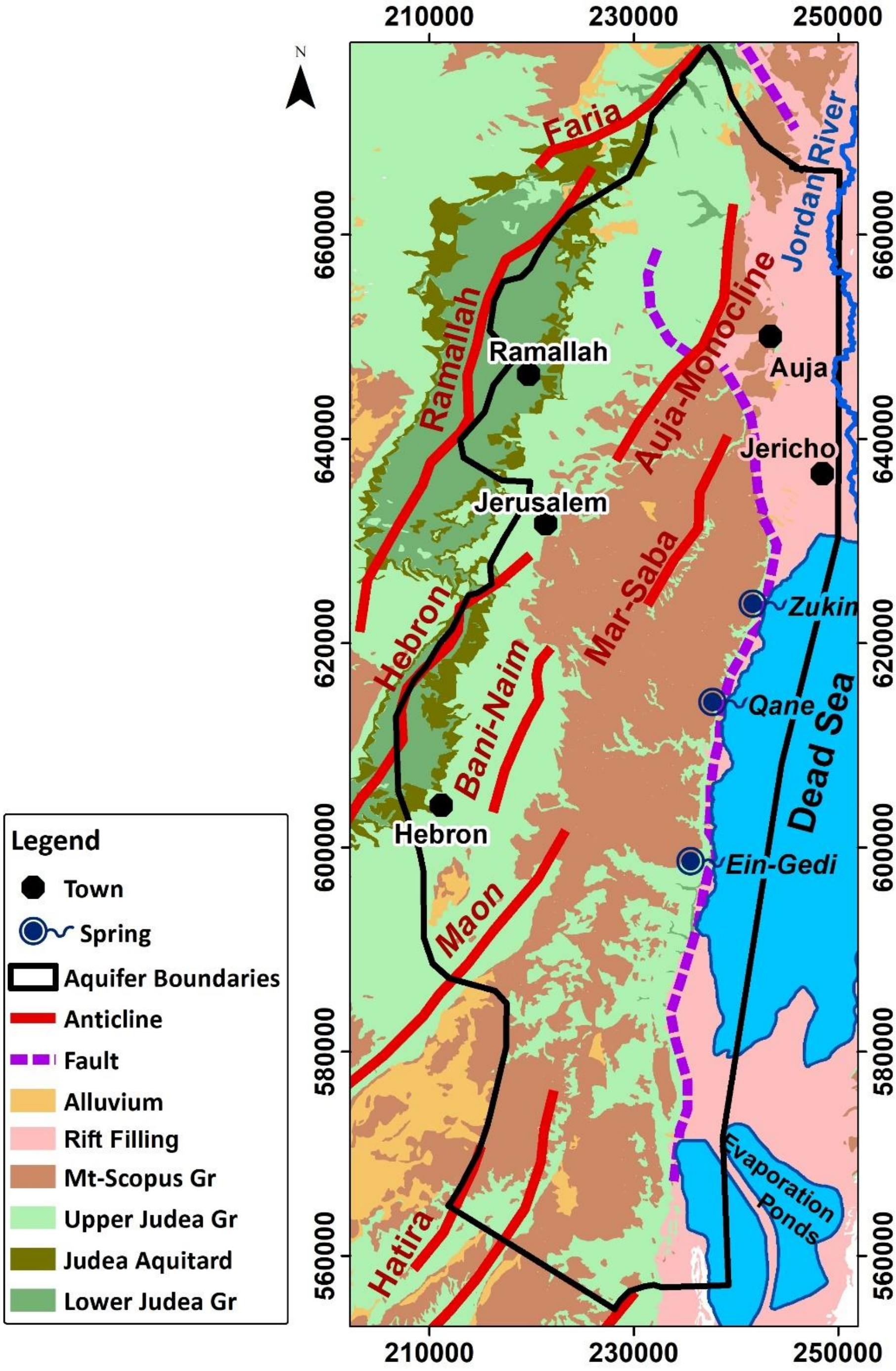

Supplementary Fig. 2. Geological map of the EMA[47]. Description of the stratigraphic units is detailed in Supplementary Fig. 3.

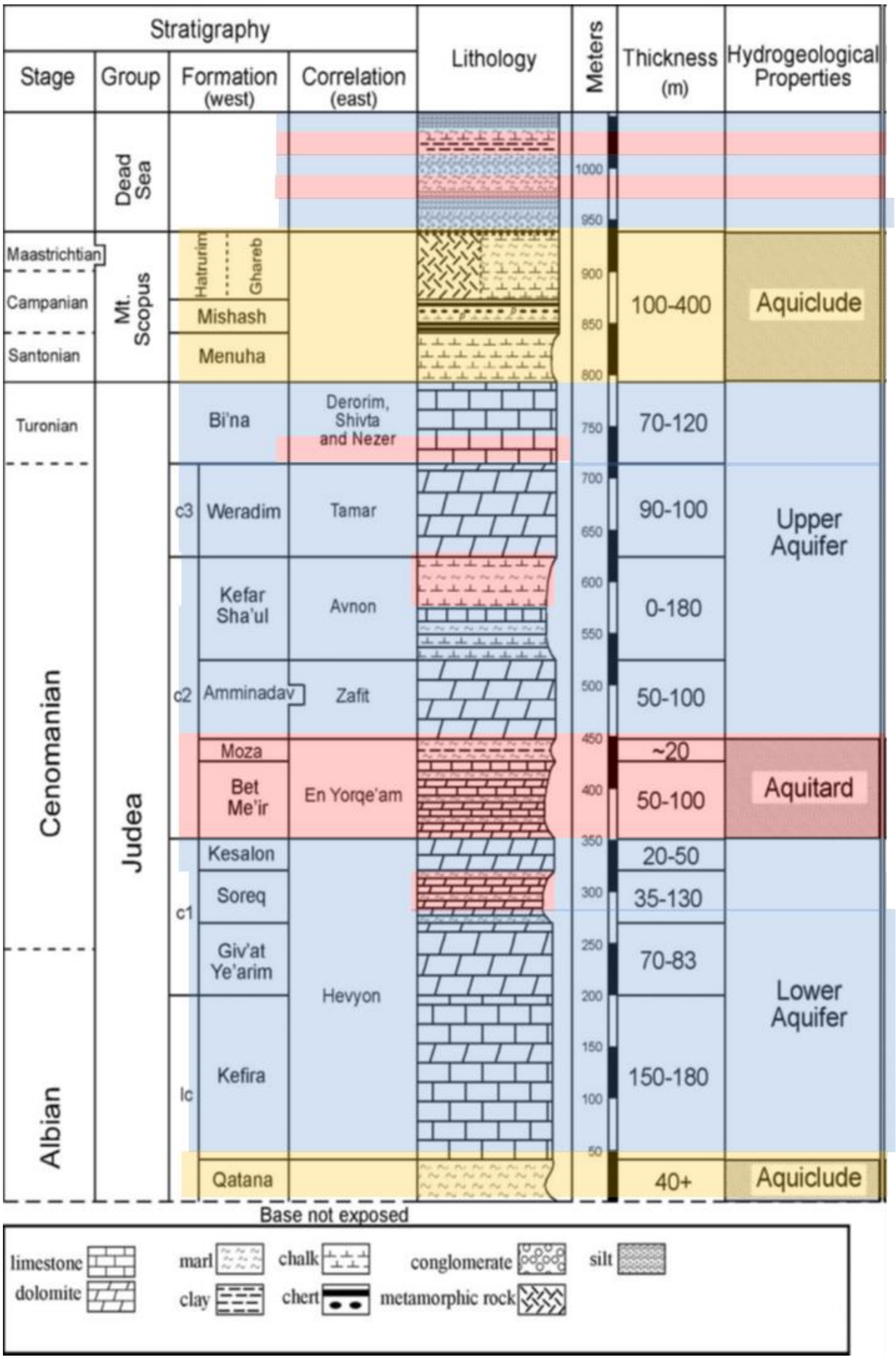

Supplementary Fig. 3. A columnar hydro-litho-stratigraphy of the studied area[29]. Colors represent aquifers, aquitards and aquicludes.

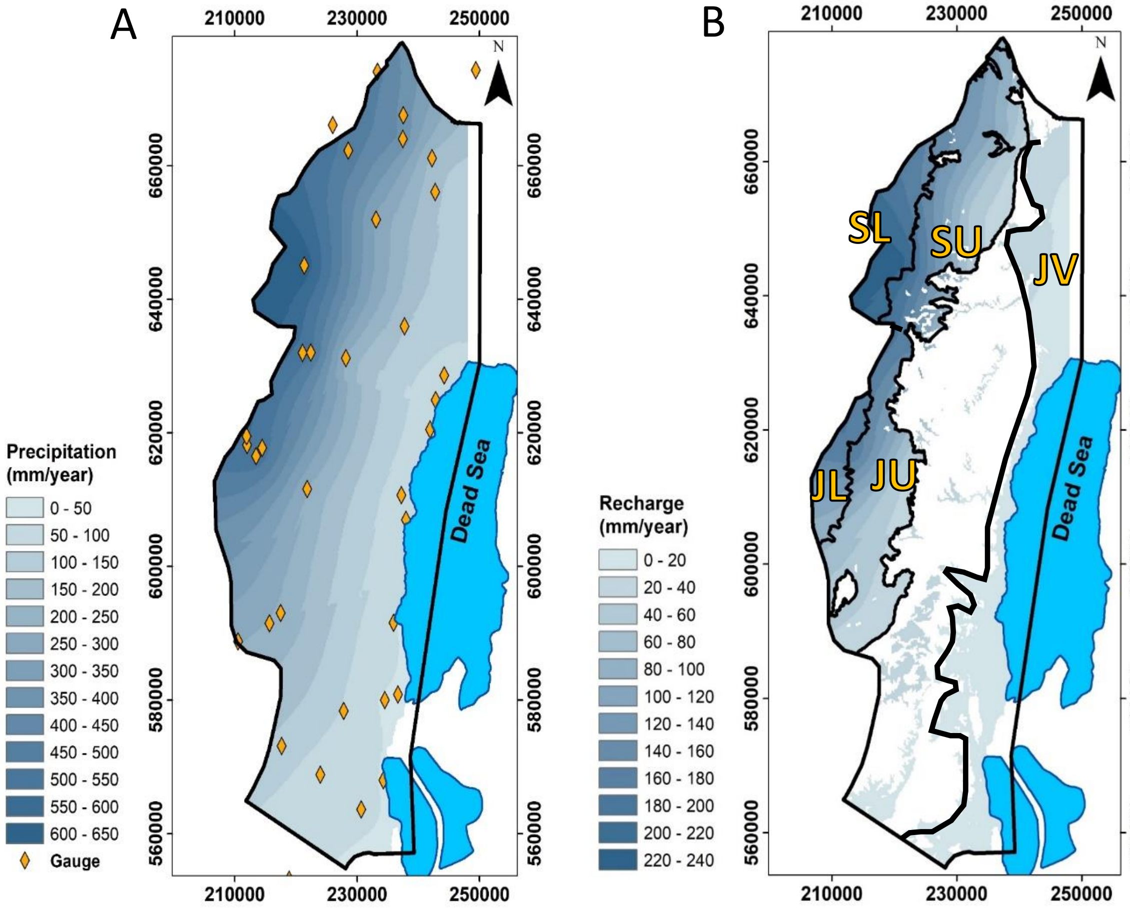

Supplementary Fig. 4. Precipitation and recharge maps. **a** – Average precipitation map (mm). **b** – Net recharge map (mm), based on well-accepted recharge coefficients[31]. Recharge occurs only in the lower and upper sub-aquifer (and occasionally in the Jordan valley, where it is not covered by the paleo lakes, see Fig. 1 and Supplementary Fig. 1). The white area represents Mt. Scopus Group outcrops (Figs. 1 and 3), which result in surface flow to the Dead Sea, sometimes as flash floods. Overall average recharge to the aquifer is ~150 mcm/y, split into five recharge zones: SL – Samaria Mountains, Lower sub-aquifer outcrops: 50 mcm/y; SU - Samaria Mountains, Upper sub-aquifer outcrops: 45 mcm/y; JL – Judea Mountains, Lower sub-aquifer outcrops: 20 mcm/y; JU - Judea Mountains, Upper sub-aquifer outcrops: 30 mcm/y; JV – Jordan Valley, Rift filling: 5 mcm/y.

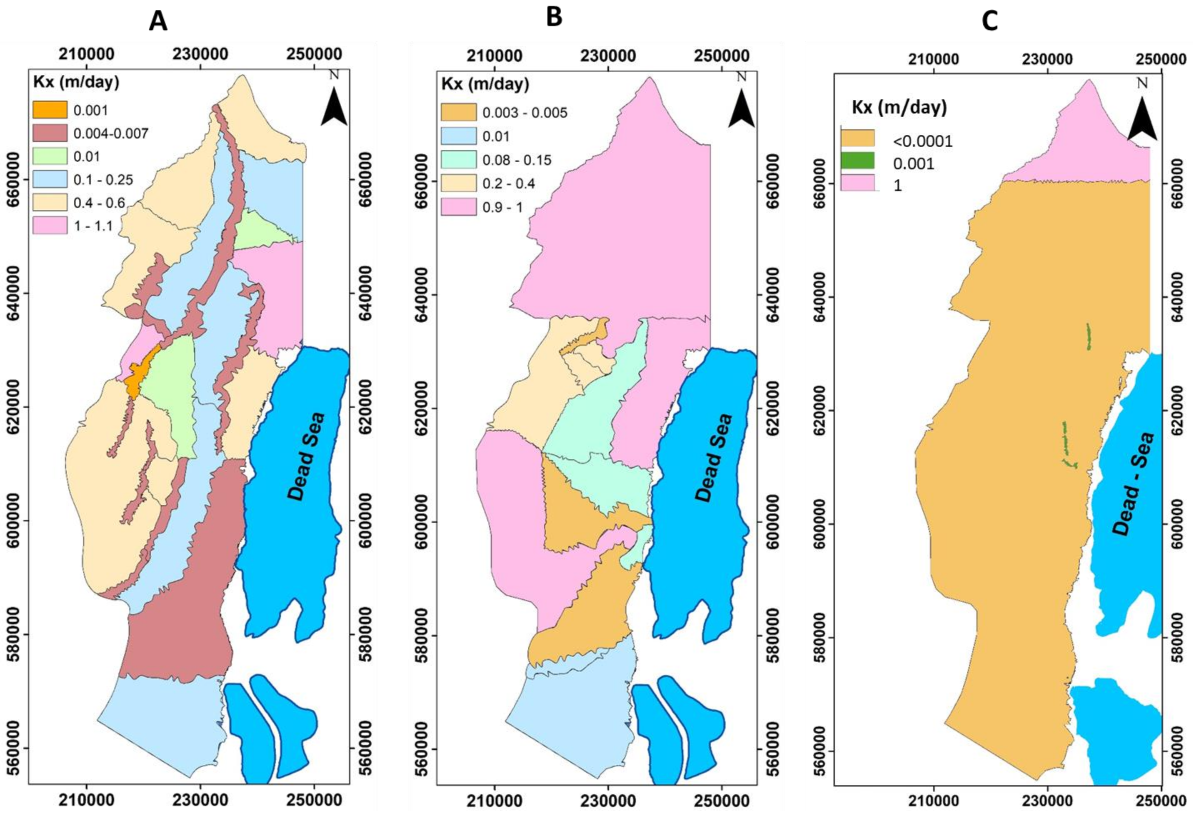

Supplementary Fig. 5. Calibrated horizontal hydraulic conductivities (Kx, m/day) of the lower and upper sub-aquifers (**a** and **b**, respectively), and of the aquitard (c).

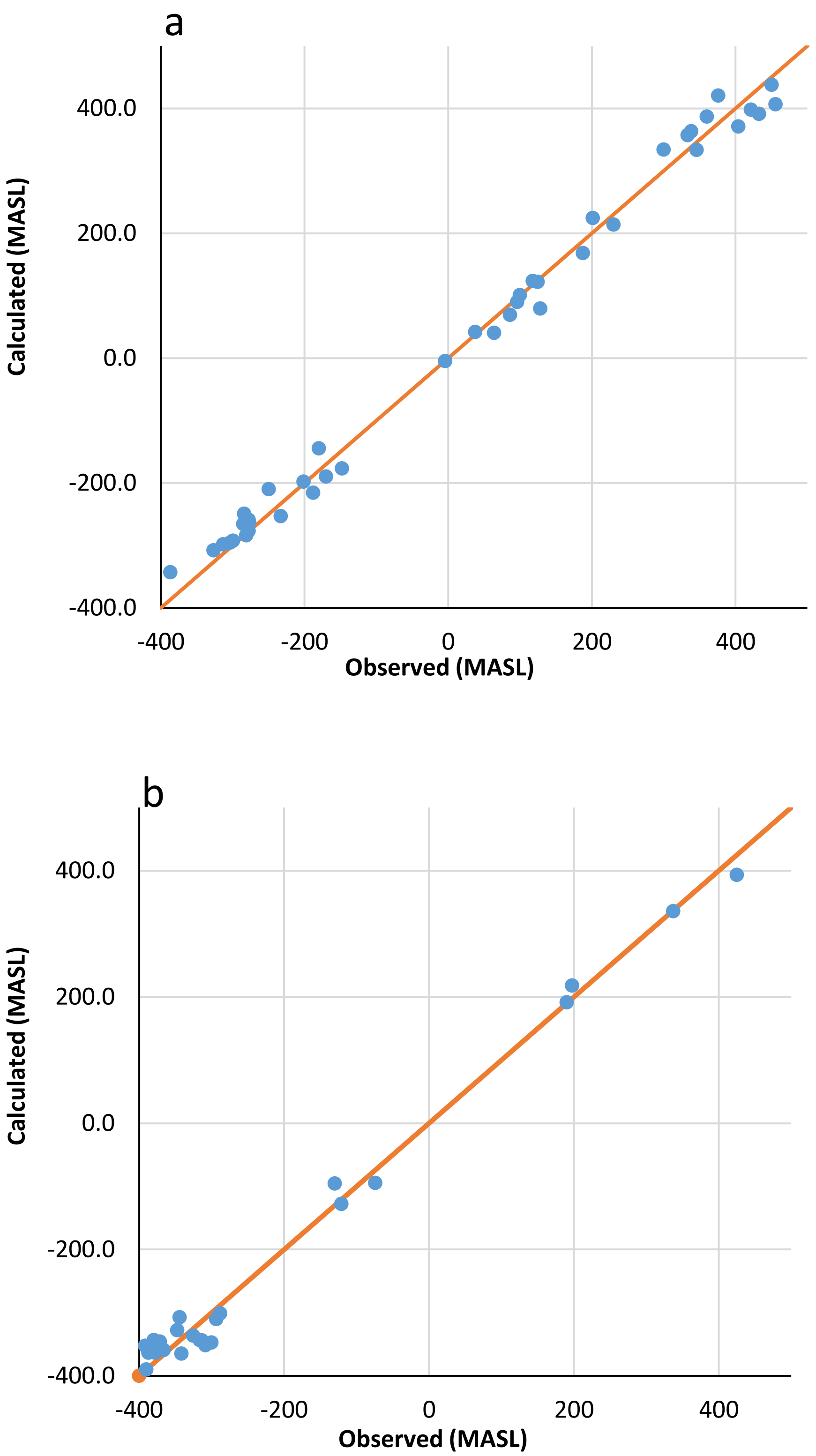

Supplementary Fig. 6. Results of the calibrated flow model. Showing calculated vs. observed hydraulic heads (masl) in wells at the lower (**a**) and upper (**b**) sub-aquifers. The red lines indicate equaled observed and calculated values.

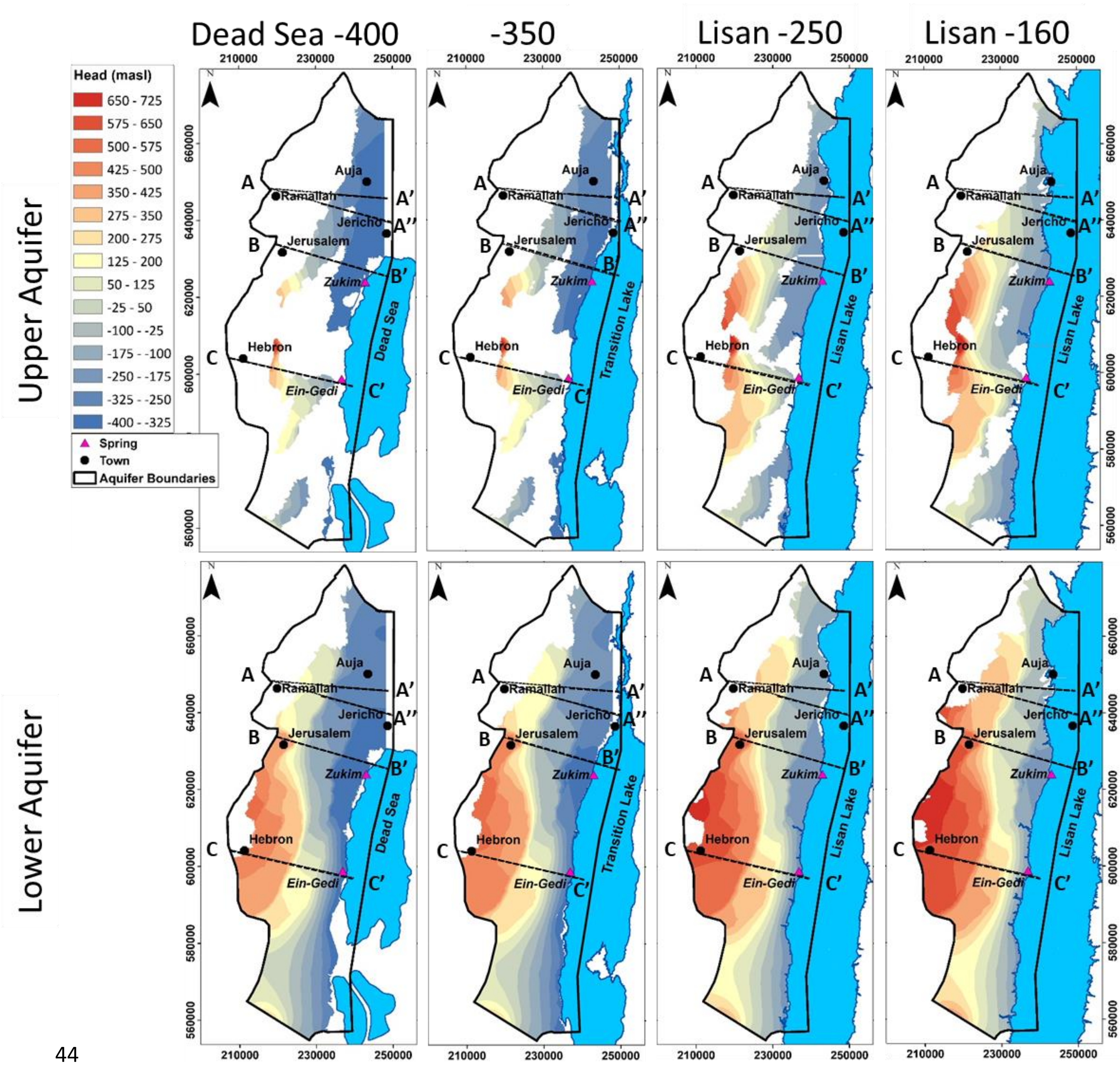

Supplementary Fig. 7. Water table maps. Eight maps of water table elevations of the upper and lower sub-aquifers (including the four maps shown in Fig. 4). Each is calculated numerically for four given lake stands: -400 mbsl of the current Dead Sea and the -350, -250 and -160 mbsl under the condition of the Lake Lisan and Transition Lake stands. Under the Dead Sea condition, the upper sub-aquifer is almost dry. Locations of vertical hydro-geological cross-sections (shown in Supplementary Fig. 9) are marked.

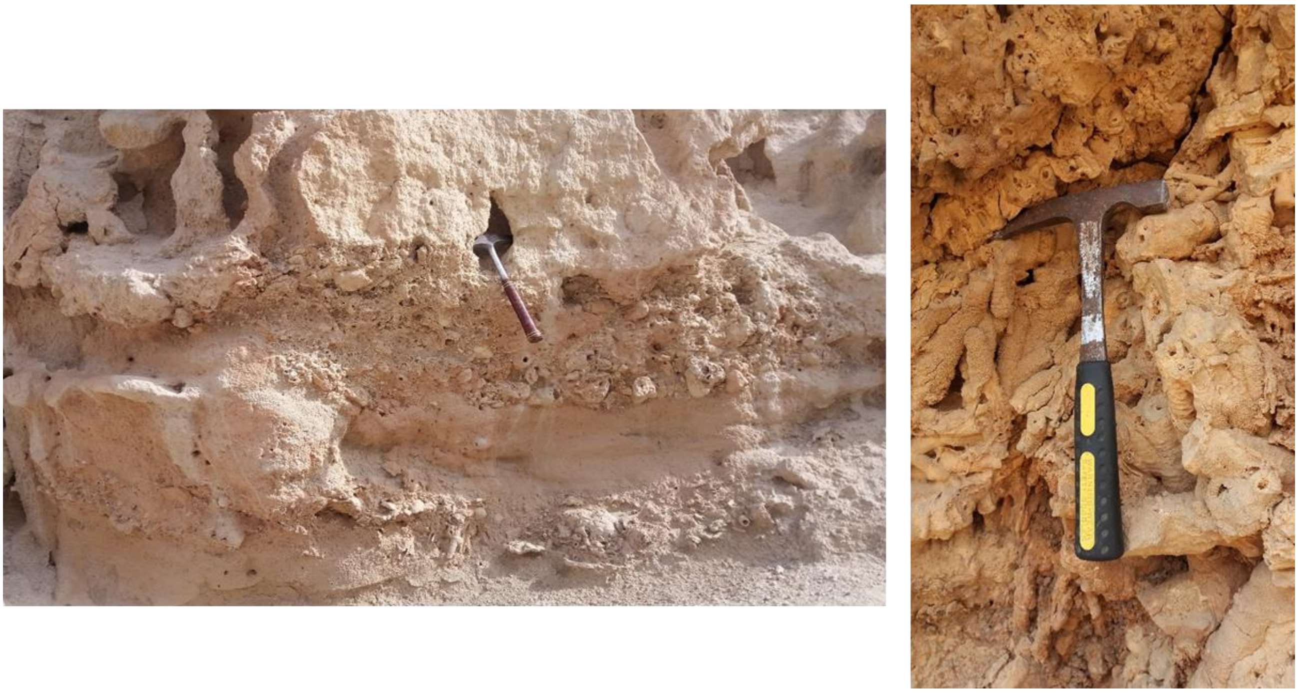

Supplementary Fig. 8. Examples of the tufa/travertine deposits of the paleo-springs in the lower Jordan Valley. The location of the image at left is near the Salibiya prehistorical sites (ITM coordinates: 243300/654500) and that at right near the Fazael prehistorical sites (ITM coordinates: 238100/662100).

58

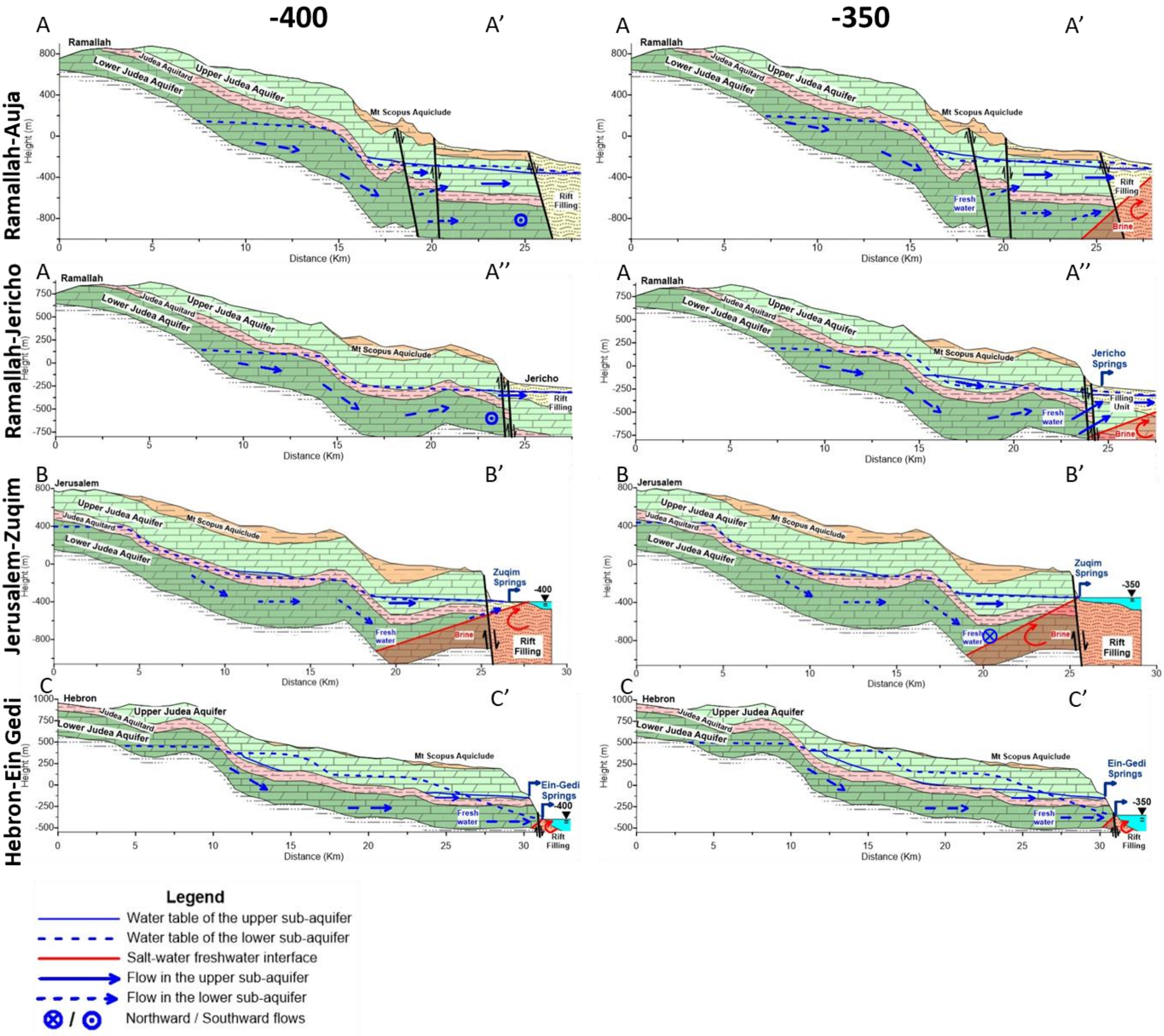
-400
-350
A
A'
A''
B
B'
C
C'
Ramallah-Auja
Ramallah-Jericho
Jerusalem-Zuqim
Hebron-Ein Gedi
Ramallah
Jerusalem
Hebron
Jericho
Upper Judea Aquifer
Lower Judea Aquifer
Judea Aquitard
Mt Scopus Aquiclude
Rift Filling
Filling Unit
Fresh water
Brine
Jericho Springs
Zuqim Springs
Ein-Gedi Springs
Height (m)
Distance (Km)
Legend
Water table of the upper sub-aquifer
Water table of the lower sub-aquifer
Salt-water freshwater interface
Flow in the upper sub-aquifer
Flow in the lower sub-aquifer
Northward / Southward flows

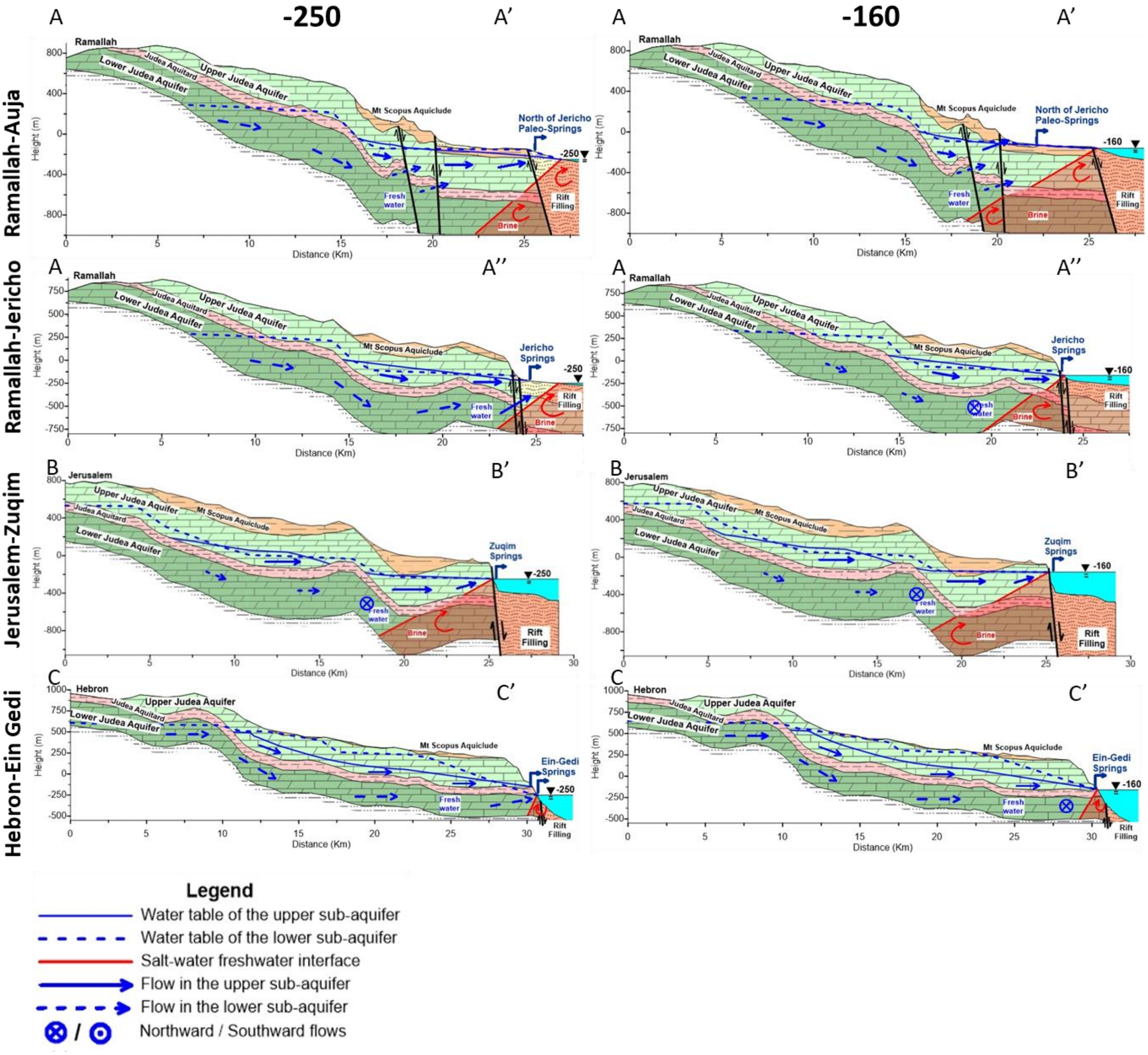

Supplementary Fig. 9. Sixteen east-west hydro-geological cross-sections, including the four that appear in Figure 5 (locations are shown in Supplementary Fig. 7). Each is calculated numerically for four lake stands. Flow lines at the upper and lower sub-aquifers and interfaces between fresh groundwater and saline lake water are marked. Due to the geological structure, high lake level is associated with blocking the discharge from the lower sub-aquifer toward the Zuqim spring zone, thus increasing the discharge in the northern spring zones (Jericho- Fazael-Auja).

Supplementary Table 1. Details on the prehistoric sites in the lower Jordan Valley. The sites are located in three main areas: (1) the earlier sites in Fazael[2,3,37,48–52], in the northern part; (2) the middle sites in Salibiya[3,53–59], in the center; and (3) the later sites in Auja/Jericho[2,3,60–63], in the south. These sites are shown on the map in Fig. 1c.

#	Period	Culture	Dates cal kBP	Climate	Fazael	Salibiya	Auja/Jericho	X	Y
1<br>2	Middle Paleolithic	Mousterian	63±4	Last-Glacial-Maximum.<br><br>High lake stand.	Fazael travertine<br>Fazael I			238120<br>240900	662090<br>662300
3	Upper Paleolithic	Atlitian	30-25		Fazael IX			237970	662430
4		Indet.			Fazael IIID			240070	662030
5<br>6	Early Epipaleolithic	Masraqan	25-20		Fazael X<br>Fazael XI			239970<br>239870	662030<br>662030
7<br>8<br>9<br>10		Early Kebaran	23-		Urkan e-Rubb II,<br>Fazael IIIB,<br>Fazael XII,<br>Talaat Zarah I			240770<br>240070<br>239450<br>240674	665030<br>662030<br>662200<br>655830
11<br>12<br>13		Late Kebaran	-18.5		Wadi Ahmar I,<br>Fazael IIIA,<br>Fazael VII			242100<br>240070<br>237770	662900<br>662030<br>662530
14<br>15<br>16<br>17<br>18<br>19	Middle Epipaleolithic	Geometric Kebaran	19/18.5-15	From Last-Glacial-Maximum to Younger-Dryas.<br><br>Decline lake level.	Wadi Ahmar I,<br>Urkan e-Rubb IV<br>Fazael IIIC,<br>Fazael VIII,<br>Talaat Zarah II,<br>El-Amri North I			242100<br>241560<br>240070<br>237770<br>240670<br>240770	662900<br>663740<br>662030<br>662530<br>655830<br>660030
20<br>21<br>22	Late Epipaleolithic	Early Natufian	15-		Fazael VI	 Salibiya XII	 <br>Jericho	237770<br>241970<br>242130	662430<br>654430<br>642060
23<br>24<br>25<br>26<br>27<br>28		Late Natufian	13.75-			Salibiya I,<br>Salibiya II,<br>Salibiya III,<br>Salibiya IV,<br>Salibiya XIV	 <br><br><br><br>Jericho	242470<br>242470<br>242570<br>242770<br>242270<br>242130	654530<br>654730<br>654730<br>654930<br>654230<br>642060
29<br>30<br>31<br>32		Final Natufian	12.7-11.6		Fazael IV	 Gilgal II<br>Gilgal VI	 <br><br>Jericho	238470<br>242800<br>242900<br>242130	662530<br>655070<br>654760<br>642060
33<br>34<br>35	Pre-Pottery Neolithic	Khiamian	11.6	Younger-Dryas.<br><br>Short Time of high lake stand.	Fazael II	 Salibiya IX	 <br>Jericho	238870<br>242300<br>242130	662330<br>654730<br>642060
36<br>37<br>38<br>39<br>40<br>41		PPNA	-10.6			Gilgal I<br>Gilgal III<br>Gilgal IV<br>Netiv Hagdud	 <br><br><br>Auja<br>Jericho,	242670<br>242470<br>242300<br>241370<br>239090<br>242130	655230<br>654830<br>654760<br>654830<br>649680<br>642060
42		PPNB	10.6-	Holocene. Decline lake stand, up to the Dead Sea.			Jericho,	242130	642060
43	Late Neolithic	Jericho IX					Jericho	242130	642060
44		Jericho VIII (Wadi Raba)					Jericho	242130	642060
45	Chalcolithic		7.7-		Fazael			241510	661610

Supplementary Table 2. Wells in the upper sub-aquifer of the EMA, and the calibration results. The "Obs Head" column is the hydraulic head (water level, masl)) observed in the wells. The "Calc Head" column is the hydraulic head in the wells that were calculated by the numerical model. The "Calc-Obs" column details the error of the calculations (residual of the calculated hydraulic head minus the observed) and "[Calc-Obs]" is this error in absolute values. In the last two rows, mean error (bias), mean absolute error and standard deviations are presented.

#	X	Y	Well Name	Obs Head (MASL)	Calc Head (MASL)	Calc-Obs	[Calc-Obs]
**1**	240770	659280	Fazael 1 (2)	-288	-301	-12	12
**2**	239300	654050	Fazael 11	-294	-310	-16	16
**3**	240900	640800	Jericho 1	-314	-344	-30	30
**4**	241400	639850	Wadi Qelt 19-14/100	-309	-351	-42	42
**5**	240720	639480	Jericho 2	-326	-336	-10	10
**6**	239110	635670	Mizpe Jericho 3	-344	-307	37	37
**7**	241700	634130	Qalia 2 Ain	-342	-365	-23	23
**8**	243500	631000	Qalia 1	-390	-390	0	0
**9**	230302	628397	Montar water	-130	-95	35	35
**10**	237587	627832	Jericho syncline	-366	-359	7	7
**11**	222740	623060	Shdema 2	198	218	21	21
**12**	223900	621900	Beit Sahur	190	192	2	2
**13**	234975	618670	Khasaba	-378	-363	15	15
**14**	220900	618300	Herodyon 1	337	336	-1	1
**15**	219460	614080	Herodyon 5	425	394	-31	31
**16**	233944	571897	Yeelim 2	-392	-352	39	39
**17**	233500	569800	Arad PP 2	-387	-363	24	24
**18**	221420	565800	Arad 5 (10)	-74	-94	-20	20
**19**	233020	562170	Nahahl Zohar 116	-372	-345	26	26
**20**	234000	562000	Nahal Zohar	-316	-343	-27	27
**21**	233925	561924	Nahal Zohar 4	-380	-343	37	37
**22**	221000	561630	Efee 13	-121	-127	-7	7
**23**	233168	560216	Nahal Hemar 2	-348	-327	20	20
**24**	232320	559752	Nahal Hemar 1	-300	-347	-47	47
					**Average**	**-0.2**	**22**
					Stdv	26	13

Supplementary Table 3. Wells in the lower sub-aquifer of the EMA, and the calibration results. See caption of Supplementary Table 2 for the column header details.

**#**	**X**	**Y**	**Well Name**	**Obs Head (MASL)**	**Calc Head (MASL)**	**Calc-Obs**	**[Calc-Obs]**
**1**	239400	668950	Gitit 1	-250	-210	40	40
**2**	239700	665200	Fazael 4	-284	-249	35	35
**3**	239340	662150	Fazael 2	-277	-263	14	14
**4**	238610	661910	Fazael 3	-233	-253	-19	19
**5**	239330	660450	Fazael 6	-278	-276	2	2
**6**	240770	659280	Fazael 1(1)	-281	-283	-3	3
**7**	238630	658750	Fazael 8	-278	-259	19	19
**8**	239060	656730	Fazael 9	-285	-265	20	20
**9**	232310	655437	Ein Samia 4	86	70	-16	16
**10**	232100	653000	Kohav Hashahar	128	80	-48	48
**11**	237350	651150	Auja 2	-148	-176	-29	29
**12**	230910	650240	Rimonim 1	100	101	1	1
**13**	236800	650050	Auja 3	-201	-197	4	4
**14**	236730	649590	Auja 4	-188	-215	-27	27
**15**	238250	646850	Jericho 5	-301	-292	7	7
**16**	239400	645150	Jericho 4	-303	-295	8	8
**17**	224110	638760	Jerusalem 11	124	122	-2	2
**18**	232700	638050	Maale Adumim 2	-170	-189	-19	19
**19**	222910	632710	Jerusalem 5	360	387	27	27
**20**	221700	630700	Jerusalem 4	433	392	-41	41
**21**	219800	628770	Jerusalem 3	422	399	-23	23
**22**	225780	628520	Abu Dis	64	41	-23	23
**23**	216880	628070	Ein Karem 16	456	407	-49	49
**24**	232040	626809	Montar Oil	-180	-144	36	36
**25**	222170	625590	Jerusalem 6	230	214	-16	16
**26**	224060	621500	Ras El Wad	201	225	24	24
**27**	220920	619330	Herodyon 2	339	364	25	25
**28**	226822	617843	Hashmura 17	118	124	6	6
**29**	220900	617500	Herodyon 3	334	358	24	24
**30**	219460	614080	Herodyon 4	376	421	45	45
**31**	217367	612391	PWA 1	450	438	-12	12
**32**	222076	608949	Rashadia 2	346	334	-12	12
**33**	229085	606538	Hashmura 11	-4	-4	0	0
**34**	224410	606179	Hashmura 48	300	334	34	34
**35**	219650	601610	Bani Naim 1A	404	371	-33	33
**36**	225080	594100	Maon 1	187	168	-19	19
**37**	233510	571905	Yeelim 4	-387	-342	44	44
**38**	232500	568870	Arad PP 3	-327	-307	19	19
**39**	233150	562100	Nahahl Zohar 5	-313	-298	15	15
**40**	215250	567200	Arad 9	96	90	-6	6
**41**	219750	565450	Arad M11	38	42	5	5
					**Average**	**1**	**22**
					**Stdv**	26	14

Supplementary Table 4. Comparison between observed and calculated annual average discharges of springs (MCM). The observed data is from the Israel Water Authority.

**Spring**	**calculated**	**Observed**
Zuqim	80	55-90
Kane—Samar	25	30-40
Toward Jordan river (north basin)	15	~10

47. Sneh, A., Bartov, Y., Weissbrodt, T. & Rosensaft, M. *Geology map of Israel, 1: 200000, 4 sheets*. (Geological Survey of Israel, 1998).

48. Bar-Yosef, O., Goldberg P & Leveson, T. Late Quaternary Stratigraphy and Prehistory in Wadi Fazael, Jordan Valley : A Preliminary Report. *Paléorient* **2**, 415–428 (1974).

49. Goring-Morris, A. N. Upper Palaeolithic sites from Wadi Fazael, Lower Jordan Valley. *Paléorient* **6**, 173–191 (1980).

50. Goring-Morris, A. N. Late Quaternary sites in wadi Fazael, lower Jordan Valley. (Hebrew University of Jerusalem, 1980).

51. Hovers, E. *et al.* The site of Urkan-E-Rub IIa: a case study of subsistence and mobility patterns in the Kebaran period in the Lower Jordan Valley. *J. Isr. Prehist. Soc.* **21**, 20–48 (1988).

52. Bar, S. *et al.* Fazael 7: A Large Chalcolithic Architectural Complex in the Jordan Valley, the 2009-2016 Excavations. *J. Isr. Prehist. Soc.* **47**, 208–247 (2017).

53. Bar-Yosef, O., Goring-Morris, A. N. & Gopher, A. *Gilgal: Early Neolithic, Occupations in the Lower Jordan Valley, the Excavations of Tamar Noy*. (Oakville, 2010).

54. Bar-Yosef, O. & Gopher, A. *An Early Neolithic Village in the Jordan Valley, Part I: The Archaeology of Netiv Hagdud*. (Peabody Museum of Archaeology and Ethnology, Harvard University, 1997).

55. Bar-Yosef, O., Gopher, A. & Goring-Morris, A. N. Netiv Hhgdud : a " Sultania " mound in the lower Jordan Valley. *Paléorient* **6**, 201–206 (1980).

56. Bar-yosef, O., Gopher, A., Tchernov, E. & Kislev, M. E. Netiv Hagdud : An Early Neolithic Village Site in the Jordan Valley. *J. F. Archaeol.* **18**, 405–424 (1991).

57. Liphschitz, N. & Noy, T. Dendroarchaeological analysis of wood remains from the Natufian and the Pre-Pottery Neolithic A sites in Gilgal. *J. Isr. Prehist. Soc.* **24**, 59–63 (1991).

58. Kislev, M. E., Bar-Yosef, O. & Gopher, A. Early Neolithic domesticated and wild barely from the Netiv Hagdud region in the Jordan Valley. *Isr. J. Bot.* **35**, 197–201 (1986).

59. Crabtree, P. J. & Campana, D. V. A Note on the First Season of Excavation at the Late Natufian Site of Salibiya I , Jordan Valley. *Paleorient* **16**, 111–114 (1990).

60. Kenyon, K. M. Earliest Jericho. *Antiquity* **33**, 5–9 (1959).

61. Kenyon, K. M. & Holland, T. A. *Excavations at Jericho volume IV: the pottery type series and other finds*. (Oxford University Press, 1982).

62. Bar-Yosef, O. The walls of Jericho : an alternative interpretation. *Curr. Anthr.* **27**, 157–162 (1986).

63. Kenyon, K. M. *The Architecture and Stratigraphy of the Tell: Excavations at Jericho*. (British School of Archaeology in Jerusalem, 1981).
